# 嵌合抗原受体T细胞的结构优化及展望

**DOI:** 10.3760/cma.j.issn.0253-2727.2021.09.013

**Published:** 2021-09

**Authors:** 川 佟, 瑶 王, 为东 韩

**Affiliations:** 解放军总医院，北京 100853 The First Medical Center, The Chinese People's Liberation Army General Hospital, Beijing 100853, China

嵌合抗原受体（CAR）以模块化方式设计，由抗原结合区和铰链组成的胞外区、跨膜区以及一个或多个共刺激分子和激活分子组成的胞内信号结构域构成。将CAR分子导入T细胞，使其具有额外的抗原特异性来重新定向靶细胞，并提供必要的激活信号来驱动T细胞的活化[1]。

CAR的抗原结合区是赋予工程化T细胞靶抗原特异性的部分，通常来源于单克隆抗体的可变轻链（VL）和可变重链（VH），经重复的甘氨酸和丝氨酸接头序列连接形成单链可变区（scFv）。为了克服单链抗体相关的结构和聚集问题，一些CAR的抗原结合区被设计成重链抗体的单可变域（VHH，也称为Nanobody）、天然配体或霉素类以取代scFv[2]–[4]。铰链（也称为间隔区）将识别抗原的胞外区连接到跨膜区。这种铰链需要一定的灵活性才能使scFv与相应的抗原结合，目前主要应用的铰链包括CD8、CD28、IgG1和IgG4等[5]–[6]。跨膜区主要是将CAR分子锚定在细胞膜上，通常来源于CD28、CD8、CD4以及可诱导T细胞共刺激分子（ICOS）等[7]。共刺激分子是CAR胞内信号结构域的重要组成部分，可以直接影响CAR T细胞的信号转导，进而演变出不同的生物学特性。目前最常用的共刺激分子来自CD28家族（CD28和ICOS）[8]–[9]和肿瘤坏死因子受体（TNFR）家族（4-1BB、CD27和OX40）[10]–[12]。CAR激活分子作为胞内信号结构域中另一个组成部分，在T细胞与靶抗原相遇时具有重要的激活作用，它主要来源于T细胞受体（TCR）CD3复合物的CD3ζ部分。然而，也有一些其他激活分子包括FcεRIγ[13]、相对分子质量70×10^3^的TCR相关蛋白激酶（ZAP70）的ζ链[14]和DAP12[15]–[16]等被用于CAR的设计。早期的第一代CAR T细胞仅有胞内激活分子而没有任何共刺激分子，由于缺乏持久性和增殖能力，所以没有显示出强大的抗瘤能力[17]–[18]。为了解决这些问题，研究人员在第二代和第三代CAR的胞内区域增加了共刺激分子，这两种CAR分别有一个和两个共刺激分子[8],[10],[19]。与第一代CAR相比，这些CAR T细胞在临床研究中表现出更强的抗瘤效能和持久性[20]–[21]。而在第四代CAR的设计中，除了胞内信号结构域外，通常还包含单独的细胞因子信号，例如IL-2、IL-12、IL-15等[22]–[23]。

目前FDA批准的所有CAR T细胞产品都是基于第二代设计理念开发的，针对CAR分子的结构优化也多集中在这一代次。在这篇综述中，我们重点讨论了CAR分子中每个组成部件对CAR T细胞信号转导和抗瘤效能的影响。

一、抗原结合区

肿瘤抗原的特异性识别是CAR T细胞治疗成功的关键，这一功能是由抗原结合区介导的。scFv作为最常用的抗原结合区，不止是简单地识别和结合靶表位，其VL和VH链之间的相互作用和接头序列的类型同样影响着CAR与靶表位的亲和力和特异性[24]。亲和力是一个特别重要的抗原结合区参数，因为它在一定程度上决定了CAR的功能输出。为了识别肿瘤细胞上的抗原，诱导CAR信号转导并激活T细胞，CAR抗原结合亲和力必须处于最佳的激活窗口。过高的亲和力会带来严重的毒性风险，而过低的亲和力则会导致不完全的T细胞活化。目前有许多优化策略旨在通过操纵亲和力来确保CAR T细胞的最佳激活。在一项基础研究中，将CD38抗体的重链与多种轻链结合构建具有不同亲和力的scFv，并对相应的CAR T细胞进行功能评测，结果显示在亲和力降低1000倍的情况下，CAR T细胞仍然有类似的抗骨髓瘤活性[25]。而用突变单链抗体构建的靶向ErbB2和EGFR的CAR也有相似的结果，亲和力较低的单链抗体提高了治疗效果[26]–[27]。最近，Ghorashian等[28]从杂交瘤中筛选出CD19特异性的单链抗体CAT，低亲和力的CAT-CAR具有与FMC63-CAR相似的表位、结构和稳定性，在基础和临床研究中均显示出强大的抗瘤效能和出色的持久性。事实上，亲和力的优化是受多方面因素影响的，不能仅考虑scFv序列，还要综合考虑其他因素，包括接头序列类型、结合表位位置、靶抗原丰度以及避免scFv相关的紧张性信号等。

目前针对scFv序列多是从鼠源化现有抗体信息中获得的，虽然其可识别人源肿瘤细胞表面抗原，但如果多次输注CAR T细胞，则需要考虑CAR的鼠源化成分所带来的免疫原性风险[29]。为避免受机体产生抗CAR T细胞的免疫反应，提高CAR T细胞的抗瘤持久性，构建人源化CAR T细胞是一个不错的优化策略。截至目前，已有一些临床研究应用人源化scFv替代鼠源序列，构建全人源化的CAR T细胞，并且在治疗血液系统恶性肿瘤中显示了良好的疗效[30]–[32]。虽然人源化CAR T细胞的初步结果显示出与鼠源化CAR T细胞相似的缓解率，但人源化CAR T细胞持续时间更长，使患者长期获益。除此之外，在一些鼠源化CAR T细胞治疗原发抵抗或复发患者中，输注人源化CAR T细胞能够获得肿瘤完全消退[33]。这凸显了人源化CAR T细胞的优越性，构建全人源化CAR分子是未来工程化T细胞发展的优选。

肿瘤细胞异质性导致肿瘤抗原动态变化是阻碍CAR T细胞治疗的重要因素[34]–[35]。为了解决肿瘤的内在抗原多样性，以及抗原表达下降或丢失的情形，靶向两个或两个以上的肿瘤抗原是一种有效的策略[36]。从概念上讲，解决抗原多样性或逃逸的最直接方法是鸡尾酒疗法，即不同特异性的CAR T细胞序贯治疗[37]。然而，为了达到GMP标准，这种策略需要在短暂的时间窗口以规定的比例混合单独制造的不同CAR T细胞，这大大增加了制作成本。此外，T细胞可以被改造成表达两种CAR分子，即双CAR[38]。这种策略赋予T细胞经典“OR布尔逻辑门”的功能，因此遇到任何一种抗原都足以激活T细胞。与相同靶点的序贯策略相比，双CAR具有更高的抗瘤活性，并且制备过程更加简便。表达两个单独的CAR分子的另一种策略是双特异性或串联CAR（TanCAR），即两个scFv都被串联设计成一个CAR分子[39]。与双CAR相比，TanCAR需要的核苷酸编码量更少，表达丰度更为均一。当两种抗原同时存在时，双scFv与靶抗原的结合可以增强免疫突触的形成，进而增强抗瘤活性[40]。然而，TanCAR的优化更为复杂，需要确定结合表位的最佳顺序和间距以避免scFv与靶表位的错配[36],[41]。最近，我们团队通过优化抗CD19和抗CD20单链抗体的顺序（构建为VHVL-VLVH或VLVH-VHVL以避免在两个单链抗体之间形成人工单链抗体）和单链抗体之间接头序列的类型，设计了一套单链双特异性CAR的优化策略[42]。与单靶向CAR相比，经CAR分子scFv区段重排筛选的CD19/CD20双特异性CAR T细胞形成类似TCR样的免疫突触结构，同时伴随大量CAR分子的聚集、纤维肌动蛋白的堆积和微管组织中心的极化等生物学效应，并且在快速钙内流的影响下，获得更快的脱颗粒效应和抗瘤效能。随后我们将CD19/CD20双特异性CAR T细胞用于治疗复发/难治性非霍奇金淋巴瘤的临床研究，显示了良好的安全性和有效性。而在一项基础研究中，Zah等[43]应用相同靶点不同来源的scFv区段构建了一套BCMA/CS1双特异性CAR T细胞的筛选体系，优化的双特异性CAR T细胞可以有效地靶向多发性骨髓瘤，显著降低肿瘤抗原逃逸的可能性。这些结果凸显了双特异性CAR胞外scFv区段优化的重要性，即使是简单的排列组合，也会引起T细胞信号转导和功能输出的差异，最终导致T细胞不同的命运转归。

二、铰链

铰链是CAR分子胞外区一个重要部件。它的作用是为抗原结合区提供灵活性以克服空间位阻，更好地识别靶抗原表位。随着研究的深入，铰链的作用也被证明比最初预期的重要，因为铰链长度和类型的差异可以影响CAR分子的表达、免疫突触的形成以及激活信号输出的强度[44]–[45]。目前常用的铰链类型来源CD8、CD28、IgG1、IgG4和IgD。Alabanza等[46]在靶向CD19的CAR T细胞中比较了CD8和CD28衍生的铰链和跨膜区的作用，基于CD8的CAR T细胞的细胞因子产生和激活诱导的细胞死亡都较低，在比较了CD8和CD28胞外域的晶体结构后，推测CAR信号强度的差异源于CD28衍生的铰链促进CAR分子的二聚化，增强了信号转导和非抗原激活的紧张性信号。此外，来源于IgG的铰链存在一个明显的缺陷，就是CH2结构域能够与Fc受体（FcγR）结合，导致CAR T细胞的非特异性激活[47]–[48]。在一些基础研究中，已经观察到IgG衍生铰链的几种有害后果，例如CAR T细胞被阻隔在肺部，激活诱导的细胞死亡导致的T细胞持续时间有限，以及由CAR T细胞非特异性激活介导的毒性[44],[49]。为了解决这些问题，一些研究团队通过突变或截断CH2结构域来降低与FcγR的相互作用，避免非抗原激活的紧张性信号，从而提高CAR T细胞的抗瘤持久性。同时将IgG衍生的铰链模块化来产生许多不同长度的铰链以优化CAR结构[47],[50]。事实上，铰链长度的优化取决于靶抗原表位的位置和靶细胞上的空间位阻水平，其中长铰链提供了更多的灵活性，允许抗原结合区更有效地捕获膜近端靶表位或复杂的糖基化抗原[51]–[52]，而短铰链在用于结合膜远端靶表位方面表现更好[53]。然而，在一项基础研究中，Qin等[54]系统地比较了IgG4-CH3和IgD铰链在不同特异性CAR T细胞中的作用，含有铰链的CAR T细胞不受靶抗原表位的影响，展现出强大的增殖、迁移和抗瘤能力。这些看似矛盾的效应，恰恰反映出铰链功能的多样性以及优化铰链的重要性。

三、跨膜区

在CAR的所有组成部件中，跨膜区可能是特征最少的区域，其主要功能是将CAR锚定在T细胞膜上。由于在许多研究中，跨膜区与其他部件（铰链或共刺激分子）经常同时更换，因此很难就它们对CAR功能的贡献做出准确的判断。然而，一些有限的数据表明，合适的跨膜区可以增强CAR的表面表达、改善CAR分子流动性和稳定性[55]–[56]。在第一代CAR结构中，CD3z衍生的跨膜区通过介导CAR分子二聚化以及与内源性TCR/CD3复合体相互作用促进T细胞的活化[55]。然而这些益处是以降低CAR的稳定性为代价的。目前在第二、三代常用的CD28和CD8衍生的跨膜区被认为是惰性受体，相对于CD3z衍生的跨膜区，大大提高了CAR的流动性和稳定性。Guedan等[56]比较了CD8和ICOS衍生的跨膜区在第三代CAR中的作用，结果显示只有当ICOS细胞内结构域直接连接到ICOS衍生的跨膜区时，第三代CAR才具有强大的抗瘤活性和持久性。最近，Wan等[57]证明了ICOS跨膜区在ICOS信号转导中的重要作用，这提示跨膜区对CAR功能的显著贡献可能是ICOS独有的。此外，在一项临床研究中，Ying等[58]在含有4-1BB共刺激分子的CD19 CAR基础上，改变CD8衍生跨膜区的长度，筛选出新型的CAR结构。这种优化的CAR T细胞在不减弱杀瘤活性的情况下减缓了增殖速率，使患者获得疾病缓解的同时大大降低了炎症因子风暴（CRS）等相关毒性。与铰链类似，跨膜区的作用还有待更多基础和临床研究证明，但重组跨膜区可能是未来CAR分子的重要优化策略，对于减少紧张性信号和提高CAR T细胞的持久性非常重要。

四、共刺激分子

在CAR T细胞治疗领域，共刺激分子活化机制的深层解析和结构优化的创新策略一直是驱动CAR T细胞突破性进化的主要动力。其不仅可以为T细胞激活提供多种信号，功能的多样性也为CAR分子提供了巨大的优化空间。具有不同共刺激分子的CAR T细胞具有不同的生物学和动力学特性，目前FDA批准的CAR T细胞产品所用的共刺激分子是CD28和4-1BB[59]。多项临床研究表明，基于CD28和4-1BB的CAR T细胞在治疗血液系统恶性肿瘤中有着相似的疾病缓解率。然而，这两种CAR T细胞的功能输出机制却不同。CD28共刺激分子诱导T细胞的快速增殖和强大的效应功能，但持久性有限[60]–[61]。相反，基于4-1BB的CAR T细胞表现出较慢的肿瘤清除动力学，但随着时间的推移而积累，最终显示出类似的抗肿瘤效果[61]。最近，Kawalekar等[62]从细胞代谢角度探讨了基于CD28和4-1BB的第二代CAR T细胞功能和持久性的区别，发现任何一种共刺激分子都能显著地重新编程CAR激活后的T细胞代谢。CD28共刺激分子诱导CAR T细胞向效应-记忆型分化，主要依赖糖酵解代谢。而4-1BB共刺激分子促进了线粒体的生物合成，增强了呼吸能力和脂肪酸的氧化代谢。此外，在抗原刺激下，含有4-1BB的CAR T细胞优先分化为中枢记忆T细胞。这一数据表明，含有CD28的CAR T细胞增加对葡萄糖的利用以获取更多能量可能是支撑其快速和强大的效应功能的因素之一，而含有4-1BB的CAR T细胞可能由于其氧化代谢能力的增强而具有更好的持久性。在另一项基础研究中，Salter等[63]利用质谱测序观察CD28和4-1BB的第二代CAR T细胞激活后的蛋白磷酸化情况。有趣的是，在抗原激活后，两种CAR引发了几乎相同的蛋白磷酸化，这表明两种CAR有类似的信号级联反应。此外，蛋白磷酸化在含有CD28的CAR T细胞中更强更快，而在含有4-1BB的CAR T细胞中要弱得多。这可能是CD28共刺激分子的PYAPP基序与淋巴细胞特异性蛋白酪氨酸激酶（LCK）的结构性关联导致的[64]，招募LCK靠近CAR细胞内信号结构域有利于信号的放大与传递。而在两项独立的基础研究中，研究者发现4-1BB共刺激分子可以招募肿瘤坏死因子受体相关因子（TRAF）来调节NF-κB信号通路进而影响CAR T细胞的信号转导和功能输出[65]–[66]。除此之外，与CD28共刺激分子相比，含有4-1BB的CAR T细胞诱导的紧张性信号较弱，进而导致CAR T细胞耗竭发生得更为缓慢[67]–[68]，这可能是含有4-IBB的CAR具有较好持久性的原因之一。

目前，已有许多研究团队通过多种途径优化共刺激分子，试图整合CD28和4-1BB共刺激分子的激活信号实现T细胞的最佳激活。在一项基础研究中，Guedan等[69]发现CD28中的单个氨基酸残基驱动T细胞耗竭并阻碍含有CD28的CAR T细胞持续存在，将CD28序列中的天冬酰胺替代苯丙氨酸可以促进CAR T细胞向Th17细胞分化（CD28突变后的YMFM基序也存在于ICOS，而ICOS对Th17细胞的发育至关重要），同时降低耗竭表型，增强抗瘤效能和持久性。在另一项基础研究中，Li等[70]将基于4-1BB的CAR胞内区域的赖氨酸突变为精氨酸来阻止CAR的泛素化，可以将内化的CAR分子重定向至T细胞表面，进一步增加4-1BB共刺激分子CAR T细胞的抗瘤持久性。最近一项研究揭示了基于CD28和4-1BB共刺激分子的CAR T细胞激活后近端调节信号的区别[71]，CD28的CAR免疫突触可以招募LCK诱导抗原非依赖性CAR-CD3ζ磷酸化，增强抗原依赖性CAR T细胞的激活；而4-1BB序列的羧基末端与胸腺选择相关蛋白（THEMIS）存在结构性关联，THEMIS本身不具有磷酸酶功能，但是其可与Src同源结构域磷酸酶-1（SHP1）磷酸酶形成复合体[72]，从而降低CAR-CD3ζ磷酸化。基于这些作用机制，研究者在4-1BB的CAR T细胞中过表达LCK以增强其抗肿瘤的动力学；而在CD28的CAR T细胞中对SHP1进行修饰，使其能够通过FKBP-FRB异二聚化过程在药理学上被招募到CAR免疫突触中，下调CAR-CD3ζ磷酸化进而降低细胞因子的分泌，这两种优化策略都被证实有助于增强CAR T细胞的信号转导和功能输出。

事实上，整合不同共刺激分子激活信号最直接的方式是将不同共刺激分子串联到一个CAR中，即第三代CAR。通常这两个共刺激分子需要来自不同的受体家族（CD28家族或TNFR家族），这样可以大大提高CAR信号的容错率。在多项基础研究中第三代CAR T细胞显示了强大的增殖能力和抗瘤持久性[73]–[74]。然而，Abate-Daga等[75]在靶向前列腺干细胞抗原（PSCA）治疗胰腺癌的基础研究中发现相比于含有CD28和4-1BB的第三代CAR T细胞，含有CD28的第二代CAR T细胞诱导更强的抗肿瘤效应，进一步研究发现可能与第二代CAR诱导类似于内源性组成型磷酸化CD3ζ的表达有关[76]。在一些临床研究中，与第二代CAR T细胞相比，第三代CAR T细胞并没有显示出更高的疾病缓解率[77]，甚至会增加CRS的发生，这可能归因于两个串联共刺激分子快速传递的重复信号。最近一些研究团队试图通过在第二代CAR T细胞中以共表达共刺激分子或受体的方式来避免这一问题。Zhao等[61]在基于CD28共刺激分子的CAR T细胞中共表达4-1BB受体，发现这种新型CAR T细胞能够诱导IRF7/IFNβ信号通路激活，展现出优越的抗瘤活性和较强的持久性。另一项研究中，Zhang等[78]筛选了12个共刺激分子，确定共表达OX40可以增强含有4-1BB的CAR T细胞的增殖能力（OX40信号上调Bcl-2家族基因来减少CAR T细胞凋亡，激活NF-κB、MAPK和PI3K-AKT信号通路促进CAR T细胞增殖），并且降低耗竭表型，维持其在肿瘤微环境中的功能。这种新型CAR T细胞在治疗B细胞淋巴瘤中显示了强大的扩增能力和抗瘤活性。不同于共刺激分子与共刺激分子的信号组合，共刺激分子与细胞因子诱导的信号组合可能更具优越性。Kagoya等[79]设计了一种新型CAR结构，抗原刺激后能够诱导细胞因子信号转导，而不是细胞因子本身。新型的CD19特异性CAR在CD28共刺激分子后插入白细胞介素2受体β链（IL-2Rβ）的胞质截短结构域和一个转录激活因子3（STAT3）的结合基序YXXQ，该基序在磷酸化后可模仿细胞因子诱导的信号转导。与传统的第二代CAR T细胞相比，这种新型CAR T细胞显示出更好的抗瘤活性和持久性。但是这种增强的CAR T细胞在转化到临床时可能会增加CRS的风险，需要在大量临床研究中来验证其安全性。

总之，CAR T细胞激活过程中共刺激分子所提供的信号对T细胞的新陈代谢、存活和效应功能起着至关重要的调节作用。每个共刺激分子都有独特的性质，微小的改动都可能对CAR T细胞的功能带来巨大影响。因此，深入了解共刺激分子的效应机制有助于为不同肿瘤类型提供精准的优化策略，以实现完全的协同作用。

五、激活分子

几乎所有的CAR结构都含有源于TCR复合体的CD3ζ激活分子，它包含三个免疫受体酪氨酸激活基序（ITAM）。ITAM是典型的免疫受体激活域，由两个Yxx（I/L）基序组成，中间有6～8个氨基酸[80]。Src家族酪氨酸激酶Lck可以磷酸化ITAM中的酪氨酸，磷酸化的ITAM可以作为激活位点招募ZAP70激酶，进一步激活后续激活T细胞连接蛋白（LAT）和磷脂酶C-γ（PLCγ）等信号分子[81]。此外，ITAM基序的数量和位置对CAR T细胞功能具有重要作用。最近，在一项基础研究中，Feucht等[82]构建了含有不同ITAM的CAR突变体（CD28共刺激分子），发现在突变CD3ζ中的第二和第三个ITAM时，CAR T细胞可在不关闭记忆程序情况下诱导强烈的效应器功能，以增强其持久的抗瘤活性。然而，James等[83]在第二代CAR（4-1BB共刺激分子）中将ITAM的数量增加到6或10个时，可以改善CAR T细胞的激活。因此，ITAM基序的数量和位置对CAR T细胞的功能有着很大影响，但这种影响并不是固定不变的，可能会受到共刺激分子种类等许多因素的影响。

此外，CD3ε、δ和γ链也可能是汽车设计的有效选择。最近，两个独立的研究团队研究结果表明将CD3ε插入到CAR结构中可以通过调节CAR信号转导来增强抗肿瘤活性，而细胞因子分泌更少，持续时间更长[84]–[85]。另一项基础研究中，Liu等[86]将抗体的抗原识别区和TCR的恒定区结合起来，构建了一种双链嵌合受体STAR-CAR，这种新型CAR T细胞不会诱发非抗原激活的紧张性信号，并且展现出更高的抗原敏感性和更强的抗肿瘤能力[86]。另一种方法是模仿内源性TCR信号。利用CRISPR-Cas9，将CAR整合到内源性TCRα链（TRAC）位点，导致其表达受内源性T细胞启动子控制。这使得CAR表达均匀，但更重要的是，可以避免CAR的紧张性信号，增强了T细胞的抗瘤能力。当暴露于抗原时，CAR被内化并重新表达，这会延迟T细胞耗竭[87]。CAR近端信号的转导依赖于CD3ζ等激活分子的激活，了解激活分子的活化机制，有助于获得可控信号强度的CAR T细胞。

六、展望

CAR T疗法是近年来最成功的转化医学之一。随着CAR T细胞制品的陆续上市，这一领域的临床和基础研究迅速扩大。然而，这种爆炸式的速度超过了基础研究所能支撑的认知，这可能会限制我们以最佳方式改进当前CAR设计的能力。因此，从机制上全面解析CAR的功能，将是设计出更成功细胞疗法的关键，需要学术界和工业界之间的紧密合作，这种伙伴关系将继续对该领域的进步起到重要作用。这篇综述重点讨论了CAR分子每个组成部件对工程化T细胞信号转导和功能输出的影响。从CAR胞外的抗原识别到胞内的信号转导再到整个T细胞的功能输出，这些组成部件参与其中，对CAR功能的贡献是不可或缺的。此外，细胞内信号转导显然是CAR胞外结构所触发的结果。scFv的亲和力、抗原结合区的结构和大小、铰链的类型和长度以及跨膜区的选择等特性都会影响信号通路的激活动力学，就像胞内共刺激分子和激活分子的选择会影响近端信号激活的特定通路一样。事实上，在整个效应过程中，这些组成部件也是相互影响相互依赖的，如scFv亲和力的优化策略不仅需要考虑scFv自身结构，还要综合考虑接头序列、铰链和跨膜区，甚至共刺激分子的影响。因此，对于单个组成部件的优化，需要放到整个CAR的效应体系中进行系统的评估。下一代CAR T细胞制品应该整合新的功能以应对现有治疗中存在的问题，无论采用哪种优化策略，都需要对CAR信号、T细胞、肿瘤细胞以及肿瘤微环境之间的相互作用有更详尽的认知，量身定制最合适的CAR T细胞以获得最佳的治疗效果。
